# Comparing Compensatory Sweating After Video-Assisted Thoracoscopic Sympathectomy: (T2-3) Versus (T2-4) Levels for Treating Palmar Hyperhidrosis

**DOI:** 10.7759/cureus.74077

**Published:** 2024-11-20

**Authors:** Peter Guirguis, Mina Girgis, Walid Abu Arab, Samir Keshk, Abdel-Meguid Ramadan

**Affiliations:** 1 Department of Cardiothoracic Surgery, Alexandria University, Alexandria, EGY

**Keywords:** compensatory sweating, hyperhidrosis, sympathectomy, thoracic surgery, video-assisted thoracoscopic surgery (vats)

## Abstract

Introduction: Primary hyperhidrosis is a disease that is characterized by excessive sweating beyond what is required to maintain the normal temperature of the body. Moreover, it has a great adverse effect on the life of the affected persons because of problems in their social lives. There are different modalities to treat primary hyperhidrosis, including medical and surgical treatment. However, in sympathectomy, there is still a lack of strong evidence regarding which level should be targeted to achieve maximum benefit with fewer complications.

Methods: This prospective clinical study was conducted at the Cardiothoracic Surgery Department, Alexandria Main University Hospital, Alexandria, Egypt, from September 2021 to Jan 2022. The study involved a total of 50 eligible consecutive patients who had bilateral primary palmar hyperhidrosis managed by bilateral, bi-portal, and tubeless thoracoscopic approach with conventional general anesthesia. Group A represents 25 patients with 13 males (52%) who had sympathetic chain cutting at the level of T2-3, and group B represents 25 patients with 15 males (60%) who had sympathetic chain cutting at the level of T2-4. The effect on palmar hyperhidrosis, compensatory hyperhidrosis, and overall patient satisfaction was assessed on the Visual Analog Scale (VAS).

Result: Ninety-eight percent of patients in both groups showed postoperative complete dryness of the hand and improvement of their symptoms. There was a statistically significant (p<0.001) difference regarding the degree of severity of compensatory sweating post-operatively between both groups. In group A, 44% of patients had compensatory sweating, while in group B, 96% of patients had symptoms of compensatory sweating with varying degrees from 1 to 5 on a VAS-Score of five degrees.

Conclusion: The level of cauterization has no significant effect on palmar hyperhidrosis dryness after cutting the sympathetic chain at level (T2-3) vs. (T2-4). However, the greater the number of levels that are cauterized, which are three levels rather than two, the more severe the compensatory sweating in this study.

## Introduction

Palmar hyperhidrosis is not an uncommon condition that is characterized by excessive sweating in the hand exceeding normal thermoregulatory needs. It apparently originates from not fully understood stimulation of the sympathetic nervous system. Its incidence is almost 2% of people worldwide, and it seems to have a genetic factor [1]. It affects males and females equally, mainly in the adolescent age. Current medical therapies for hyperhidrosis are classified into several groups, including topical, oral, injectable medications, medical devices, and laser and energy‐based device therapies (miraDry, ultrasound therapy, fractional microneedle radiofrequency, and laser therapy) [2]. The current surgical modality is video-assisted thoracoscopic sympathectomy, which is linked with great success and low morbidity rate when applied in the treatment of hyperhidrosis [3]. Surgery is considered the best modality for its treatment. It is achieved through the interruption of the sympathetic chain and ganglia via conventional sympathectomy or the cutting of the sympathetic trunk via sympathectomy at different levels depending on the sweaty areas [1].

The thoracic chain contains a ganglion for every spinal nerve; the first one regularly unites with the inferior cervical ganglion to make the stellate ganglion, which, if injured, can cause Horner syndrome. Sympathetic fibers, which are branches of the sympathetic chain, are directed to the skin accompanied by each of the thoracic spinal nerves [3,4].

Hyperhidrosis can be divided into primary, which is idiopathic, or secondary, which is due to an underlying cause. Primary hyperhidrosis is involving the palms, axillae, soles, and the groins. However, molecular pathways regulating sweat gland function, such as the Wnt, Sonic Hedgehog (Shh), and Notch pathways, may play a crucial role in its pathogenesis [5]. These pathways regulate cellular processes like proliferation and differentiation, potentially leading to increased sensitivity or a number of eccrine glands, which are hyperactive in primary hyperhidrosis [5]. Hyperhidrosis may also alter skin microcirculation, leading to capillary dysfunction and increased vasoconstriction. It stops during sleep and increases with anxiety, pain, stress, and caffeine intake, but it does not change with the different seasons; the diagnosis is made mainly by exclusion [6].

Compensatory sweating is the most reported and unpredictable adverse effect, with severe symptoms reported in nearly 74.5% of patients. Gustatory sweating is less commonly reported. Horner syndrome after sympathectomy has markedly decreased in ongoing surgical practice [7]. The optimal postoperative results are highly dependent on the selection of the correct patient for the suitable procedure [8].

There aren't any established contraindications to sympathectomy, but patients who are obese and overweight don't seem to respond well to sympathectomy and are at a higher risk of developing significant compensatory sweating [9]. The aim of the study is to detect the effect of sympathectomy level on palmar hyperhidrosis and compensatory sweating.

## Materials and methods

A prospective study was conducted at the Cardiothoracic Surgery Department, Alexandria Main University Hospital, Alexandria, Egypt, from September 2021 to Jan 2022. Institutional Review Board ethical approval was granted for outcome analysis in this study (IRB 00012098, August 19, 2021), and informed consent was obtained from all patients for the relevant surgical procedures, as well as anonymized inclusion into this study. All methods of this study were conducted following the relevant regulations for working with human subjects and the Declaration of Helsinki [10]. All methods of this study were conducted following the relevant regulations for working with human subjects and the Declaration of Helsinki. Allocation was randomized and numbered using the closed envelope technique, and masking was single-blinded. All included patients signed an informed consent for the operation. Approval of the medical ethics committee at Alexandria Faculty of Medicine was obtained.

Intraoperatively, after the thoracoscope was introduced, the sympathetic chain was identified as lying under the pleura and crossing perpendicularly to the head of the ribs. The ribs were used as landmarks to locate the desired levels to work on (R2-2nd rib, R3-3rd rib, and R4-4th rib). Under video assistance, the parietal pleura over the correspondent level was opened using hook cautery through the 5 mm trocar.

**Figure 1:**
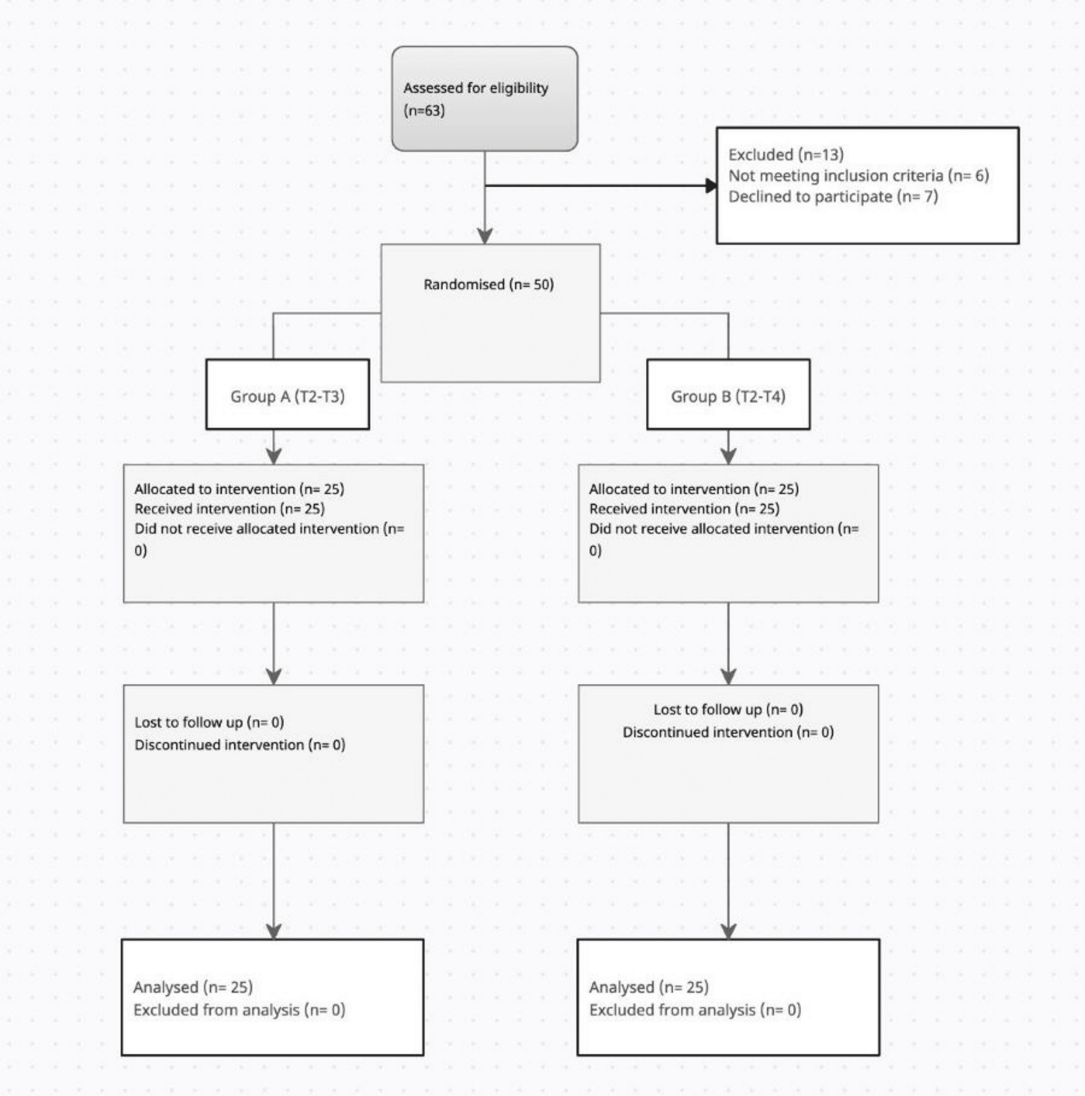
CONSORT flowchart for the search strategy

The patients were alllocated into two groups as per the following search strategy (Figure 1).

In Group A, the sympathetic chain at levels T2-3 was cut using either a hook or a Maryland grasper. The segment of the sympathetic chain at these levels was successfully acquired. Following this, cauterization was performed over the upper border of the 2nd and 3rd ribs laterally, extending approximately 2-3 cm and including the nerve of Kuntz, using a Maryland or hook cautery to minimize the risk of recurrence.

In Group B, the sympathetic chain was cut at levels T2-4 using either a hook or a Maryland grasper, with segments at T2, T3, and T4 successfully excised. Subsequently, cauterization was performed along the upper borders of the 2nd, 3rd, and 4th ribs, including the nerve of Kuntz, extending laterally for approximately 2-3 cm. This was achieved using a Maryland or hook cautery to minimize the likelihood of recurrence.

Our primary indicator for evaluating the effect of endoscopic sympathectomy on hyperhidrosis was mainly subjective. The effect on palmar hyperhidrosis was assessed by measuring hand dryness on a 10-point Visual Analog Scale (VAS) at three time intervals: one month, six months, and one year in both groups. Compensatory hyperhidrosis was evaluated using a 5-point VAS scale at the same intervals: 1 month, 6 months, and 1 year. Overall patient satisfaction with the procedure was also assessed on a 5-point VAS at the same three intervals. Additionally, we recorded the time required for the patient to return to daily life and the duration for which analgesia was needed.

In the statistical analysis, data were fed to the computer and analyzed using IBM SPSS software package version 20.0. (Armonk, NY: IBM Corp) Qualitative data were described using numbers and percentages. The Shapiro-Wilk test was used to verify the normality of distribution. Quantitative data were described using range (minimum and maximum), mean, standard deviation, median, and interquartile range (IQR). The significance of the obtained results was judged at the 5% level. The Chi-square test, Fisher’s Exact test and Mann Whitney test have been used.

## Results

Our sample included a total of 50 eligible patients divided into two equal groups that were statistically matched. In Group A, the mean age was 17.80±4.89 years, with 13 participants (52%) being male. In Group B, the mean age was 19.36±2.91 years, with 15 participants (60%) being male (Table 1).

**Table 1:** Comparison of the two study groups based on demographic data

Demographic data	Group A (n=25)	Group B (n=25)	Test of sig.	p-value
Sex	-	-	-	-
Male	13 (52%)	15 (60%)	x^2^=0.325	0.569
Female	12 (48%)	10 (40%)		
Age (years)	-	-	-	-
Min-Max	12.0-25.0	13.0-22.0	t =1.370	0.844
Mean±SD	17.80±4.89	19.36±2.91		
Median (IQR)	19.0 (13.0-20.0)	21.0 (18.0-22.0)		

The duration of surgery in group A ranged from 23 to 40 minutes (32.75±3.12), while in group B, it ranged from 39 to 51 minutes (44.53±4.02). No intraoperative thoracoscopic sympathectomy complications were encountered in both groups. Regarding the postoperative complications, excluding compensatory sweating, there was no complication in Group A. There was only one patient with wound infection (4%) in group B, which was managed by frequent dressing with betadine and bivatracin spray (Table 2).

**Table 2:** Comparison of the complications between the two study groups

Complications	Group A (n=25)	Group B (n=25)	x^2^	p-value
Intraoperative	0 (0%)	0 (0%)	-	-
Post-operative	-	-	-	-
Nil	25 (100%)	24 (96%)	1.020	^FE^p= 1.000
Wound infection	0 (0)	1 (4%)		

Patients in both groups were asked to evaluate the palmar hyperhidrosis subjectively on a VAS score of 10 degrees preoperatively and postoperatively. Preoperatively, the mean of the score in group A (9.12 ± 0.78), while group B was (9.08±0.76). Postoperatively, the VAS score in the two groups was followed up after one, six, and twelve months.

In patients included in group A, the mean postoperative VAS score of palmar hyperhidrosis at one month was (1.20±0.41), and was (1.08±0.28) after 6 months. It was the same even after one year of surgery, where it was (1.08±0.28). In group B, the mean of the VAS score after one month of surgery was (1.12±0.33) at one month, (1.04±0.20) at 6 months, and it was (1.0±0.0) by the end of the first year postoperatively. There was no significant statistical difference between both studied groups at any of the time intervals (Table 3).

**Table 3:** Comparison of the two study groups according to severity of palmar hyperhidrosis on Visual Analog Scale score

Palmar hyperhidrosis (PH) Visual Analog Scale (VAS) 10	Group A (n=25)	Group B (n=25)	U	p-value
Preoperative	-	-	-	-
Min-Max	8.0-10.0	8.0-10.0	303.0	0.844
Mean±SD	9.12±0.78	9.08±0.76		
Median (IQR)	9.0 (9.0-10.0)	9.0 (9.0-10.0)		
1 Month	-	-	-	-
Min-Max	1.0-2.0	1.0-2.0	287.50	0.445
Mean±SD	1.20±0.41	1.12±0.33		
Median (IQR)	1.0 (1.0-1.0)	1.0 (1.0-1.0)		
6 Months	-	-	-	-
Min-Max	1.0-2.0	1.0-2.0	300.0	0.556
Mean±SD	1.08±0.28	1.04±0.20		
Median (IQR)	1.0 (1.0-1.0)	1.0 (1.0-1.0)		
1 Year	-	-	-	-
Min-Max	1.0-2.0	1.0-1.0	287.50	0.153
Mean±SD	1.08±0.28	1.0±0.0		
Median (IQR)	1.0 (1.0-1.0)	1.0 (-)		

Patients were assessed subjectively with a visual analog scale of five degrees at three time intervals: one, six, and twelve months postoperatively for the occurrence of compensatory sweating in both groups.

In group A, the mean of VAS score of five degrees was (1.52±0.65) at the three-time intervals without any change, while in group B, the mean of VAS score was (2.96±1.10) at the three-time intervals without any change. The VAS score between both groups showed a significant statistical difference between both groups with a p-value less than 0.001 (Table 4). Compensatory sweating was observed at the following sites: the thigh in 7 participants (28%) from Group A and 11 participants (44%) from Group B; the back in 2 participants (8%) from Group A and 7 participants (28%) from Group B; the abdomen in 2 participants (8%) from Group A and 5 participants (20%) from Group B; and gustatory sweating was reported in none (0%) of Group A compared to 1 participant (4%) in Group B.

**Table 4:** Comparison of the postoperative compensatory sweating on Visual Analog Scale scores between the two study groups * p-value of <0.001 showed a significant statistical difference between both groups at three time intervals (one, six, and twelve months).

Post-operative compensatory sweating (CS) Visual Analog Scale (VAS) 5	Group A (n=25)	Group B (n=25)	U	p-value
1 Month	-	-	-	-
Min-Max	1.0-3.0	2.0-5.0	88^*^	<0.001^*^
Mean±SD	1.52±0.65	2.96±1.10		
Median (IQR)	1.0 (1.0-2.0)	3.0 (2.0-4.0)		
6 Months	-	-	-	-
Min-Max	1.0-3.0	2.0-5.0	88^*^	<0.001^*^
Mean±SD	1.52±0.65	2.96±1.10		
Median (IQR)	1.0 (1.0-2.0)	3.0 (2.0-4.0)		
1 Year	-	-	-	-
Min-Max	1.0-3.0	2.0-5.0	88^*^	<0.001^*^
Mean±SD	1.52±0.65	2.96±1.10		
Median (IQR)	1.0 (1.0-2.0)	3.0 (2.0-4.0)		

Patient satisfaction was assessed by a VAS score of five degrees at three time intervals: one, six, and twelve months postoperatively for patients in each group. Patients were asked about their overall satisfaction on a VAS score of five degrees, where one degree is reported for not-satisfied, and five corresponds to well-satisfied patients. In group A, the mean of the VAS score reported at the three-time intervals was (4.88±0.44); while in group B it was (4.04±1.24). There was a significant statistical difference between both groups (Table 5).

**Table 5:** Comparison of the two study groups according to patient satisfaction on Visual Analog Scale score * p-value of <0.001 showed a significant statistical difference between both groups at three time intervals (one, six, and twelve months).

Patient satisfaction Visual Analog Scale (VAS) 5	Group A (n=25)	Group B (n=25)	U	P value
1 Month	-	-	-	-
Min-Max	3.0-5.0	1.0-5.0	184.5^*^	<0.001^*^
Mean±SD	4.88±0.44	4.04±1.24		
Median (IQR)	5.0 (5.0-5.0)	5.0 (3.0-5.0)		
6 Months	-	-	-	-
Min-Max	3.0-5.0	1.0-5.0	184.5^*^	<0.001^*^
Mean±SD	4.88±0.44	4.04±1.24		
Median (IQR)	5.0 (5.0-5.0)	5.0 (3.0-5.0)		
1 Year	-	-	-	-
Min-Max	3.0-5.0	1.0-5.0	184.5^*^	<0.001^*^
Mean±SD	4.88±0.44	4.04±1.24		
Median (IQR)	5.0 (5.0-5.0)	5.0 (3.0-5.0)		

## Discussion

There is no established guideline for the optimal level at which sympathectomy should be performed to achieve the best treatment outcomes with minimal complications. Thoracoscopic sympathectomy provides a safe, permanent, and rapid end to the excessive sweating problem. It is performed under general anesthesia by traditional ETT (endotracheal tube) and CO_2_ or double lumen ETT and cutting sympathetic chain [11]. 

Our study involved 50 patients with a main complaint of primary palmar hyperhidrosis. None of them had tried any medical treatment before. The majority of the patients were males (n=28, 56%). The incidence of palmar hyperhidrosis in this study was slightly higher in males than that was reported by Cerfolio RJ et al., who suggested that the incidence is mostly equal, but more females seek medical consultation as they are more sensitive to stressful events that can exacerbate the hyperhidrosis [3]. This slightly higher incidence of palmar hyperhidrosis in this study was attributed to the fact that the majority of patients sought medical advice for the reason of their manual work and not for social reasons, and most of those workers were males who are involved more in manual work.

Haider and Solish stated that palmar hyperhidrosis started early at the age of 13 [12]. In our study, the age ranged between 12 and 25, with the mean age in group A as 17.80±4.89 and in group B as 19.36±2.91. Regarding age, there was no significant statistical difference between both groups as they have the same disease, and they were blindly distributed in each group. Moreover, our findings were consistent with those reported by Strutton et al. [13].

Preoperatively, the VAS score regarding the severity of palmar hyperhidrosis showed a mean of 9.12±0.78 in patients included in group A, and it was 9.08±0.76 in patients included in group B. Moreover, 10 patients in group A (40%) and 12 patients in group B (48%) showed 10 degrees on the VAS score. Post sympathectomy, the mean of the VAS score of ten degrees evaluating the severity of palmar hyperhidrosis was 1.20±0.41 and 1.12±0.33 for both groups A and B, respectively, at one-month intervals post-surgery. This reflects the high degree of efficacy of VATS-sympathectomy in the management of primary palmar hyperhidrosis. Although the means of VAS score decreased over time follow-up, as reported at 6 and 12-month intervals in patients of both groups, this decrease was slight.

The VAS score showed a slight improvement over the later times at 6 and 12 months in both groups, which was insignificant statistically compared to the one recorded at the first-month follow-up interval. Many researchers have studied the level at which sympathectomy can be done and have found that it can result in efficient and satisfactory results. Abu Arab et al., in their study, showed a dramatic postoperative decrease in the degree of severity of palmar hyperhidrosis, which was assessed by a VAS score of ten degrees in which zero represents no more sweating after sympathetic chain cauterization at the level T2-T4 [14]. The results showed a mean of 0.095±0.296 with success rate above 97.5%. At the level of T3-T4, Huang et al. cut the sympathetic chain and showed 100% effective improvement in the symptoms [15].

In our study, the success rate after cauterizing level T2-T3 was above 96%, where 24 patients experienced a VAS score of 1, while at level T2-T4, it showed improvement in the symptoms of 25 patients (100 %). This slight difference in success rate, which is illustrated by patient satisfaction on the VAS score scale, was in favor of the level of T2-T4 and can be attributed to slight variation in chain anatomy or the presence of branches at lower levels that have been cauterized during extended procedures at lower levels. However, the slight success rate difference between the two groups was found to be statistically insignificant.

Compensatory sweating is the most annoying complication after sympathectomy, which results in excessive sweating at other parts of the body such as back, thigh, abdomen and buttock. It is common in around 30-70 % [16]. Wei et al. reported that compensatory sweating occurred in 37.6% of T3 sympathectomy level patients and in around 47.7% of T4 sympathectomy level patients [16]. This result is inconsistent with most of the published results literature, suggesting that T3-4 may be more effective in the treatment of palmar hyperhidrosis with a lower incidence of postoperative compensatory sweating [17-18]. Some authors reported that minimizing the levels of interruption of the sympathetic chain is limiting the incidence and severity of compensatory sweating; however, others reported that it has no effect on compensatory sweating [19-21].

Abu Arab et al. assessed the compensatory sweating in 518 patients following sympathectomy at the level from T2-T4 [14]. It was assessed using a VAS score of ten degrees, where zero represented no compensatory sweating and 10 degrees represented the most severe form. The study showed that 3.9 % of patients suffered from grade 0-4 compensatory sweating after 6 months, which was considered a mild degree of compensatory sweating. The mean of the compensatory sweating was 2.21± 0.926 after 6 months.

In this study, the patients included in group A showed a range of VAS-Score regarding compensatory sweating between 1 and 3 with a mean of 1.52 ± 0.65 and insignificant changes over time. About 44% of patients had compensatory sweating, and only 8% experienced a VAS score of 3. On the other hand, group B patients showed 96% symptoms of compensatory sweating with varying degrees from 1 to 5, and there were 8% suffering from a severe form of compensatory sweating by selecting 5 on VAS-Score. This difference between the two groups may be explained by the number of levels that had been cauterized. The most common sites described by the patients were thigh (51.43 %), back (25.71%), abdomen (20%), and gustatory sweating (2.8%).

Aoki et al. demonstrated in their results that the degree of severity of compensatory sweating is decreased by sympathectomy performed at one level compared to multiple levels [22]. Kara and colleagues mentioned that mild-to-moderate compensatory sweating was found in 41 patients (78%) while disturbing severe compensatory sweating was found in 4 patients (7%) of their study sample [23].

Patient satisfaction depends mainly on two main factors. First, the assessment of the symptoms was done on a VAS score of 10 degrees after surgery, and second, the severity of compensatory sweating was assessed at three time intervals during postoperative follow-up. Chang et al. showed that the severity of compensatory sweating following sympathectomy is strongly inversely correlated to the degree of patient satisfaction, which validates our results [24]. 

In our study regarding patient satisfaction, there were no statistical changes over time, although the numbers showed a statistically significant difference between both groups. Patient satisfaction was subjectively assessed by VAS score on a scale of 5 degrees, whereas five represents that the patient is well satisfied. In group A, patients who chose score 5 were 23 (92%), and 2 patients scored 3 and 4. In group B, the patients who chose 5 were 13 (52%), and one patient chose one degree on VAS-Score. This patient, unfortunately, stated that it was better not to do surgery. The difference denotes that group A patients were more satisfied than patients in group B.

The duration of surgery in group A ranged between 23 and 40 minutes, with a mean of 32.75±3.12. In group B, it ranged from 39 to 51 minutes with a mean of 44.53±4.02. The difference in duration between the two groups is explained by more levels to be cauterized in group B. On the other hand, the difference in duration of surgery between patients in the same group was most likely due to the difference in BMI, which takes more time in obese patients. Hospitalization time post-operatively ranged from 4 - 8 hrs. All the patients were discharged the same day after performing a chest X-ray. Abu Arab et al. reported a range from 4 to 48 hours. Pain in all patients was controlled by the synergistic effect between paracetamol and NSAIDs [14]. Therefore, all patients in both groups returned to their daily activities the day after surgery. There were no intraoperative complications. However, postoperative complications were encountered in one patient from group B, where it was in the form of superficial wound infection, which was controlled by frequent dressing.

The limitation of our study is related to the sample size and diversity, as a small or homogenous sample may limit the generalizability of the findings. Additionally, the assessment of compensatory sweating primarily relies on subjective patient-reported outcomes.

## Conclusions

This study confirms that endoscopic thoracic sympathectomy is an effective and safe technique for the treatment of primary palmar hyperhidrosis. The level of cauterization has no significant effect on palmar hyperhidrosis dryness. However, in this study, the greater the number of levels cauterized, the more severe the compensatory sweating. Patient satisfaction is inversely correlated with the severity of compensatory sweating. Neither T2-T3 nor T2-T4 levels show a significant difference in complications or postoperative hospital stay for treated patients. These findings can guide reducing the levels of sympathectomy to achieve optimal satisfaction with fewer complications.
